# Prediction of Improved Antimalarial Chemoprevention with Weekly Dosing of Dihydroartemisinin-Piperaquine

**DOI:** 10.1128/AAC.02491-16

**Published:** 2017-04-24

**Authors:** Jesmin Permala, Joel Tarning, François Nosten, Nicholas J. White, Mats O. Karlsson, Martin Bergstrand

**Affiliations:** a Department of Pharmaceutical Biosciences, Uppsala University, Uppsala, Sweden; b Faculty of Pharmacy, Universiti Teknologi MARA (UiTM), Puncak Alam Selangor, Malaysia; c Mahidol-Oxford Tropical Medicine Research Unit, Faculty of Tropical Medicine, Mahidol University, Bangkok, Thailand; d Centre for Tropical Medicine and Global Health, Nuffield Department of Medicine, University of Oxford, Oxford, United Kingdom; e Shoklo Malaria Research Unit, Mae Sot, Thailand

**Keywords:** dihydroartemisinin-piperaquine, IPT, malaria, nonadherence, pharmacodynamics, pharmacokinetics, weekly dosing

## Abstract

Intermittent preventive treatment (IPT) is used to reduce malaria morbidity and mortality, especially in vulnerable groups such as children and pregnant women. IPT with the fixed dose combination of piperaquine (PQ) and dihydroartemisinin (DHA) is being evaluated as a potential mass treatment to control and eliminate artemisinin-resistant falciparum malaria. This study explored alternative DHA-PQ adult dosing regimens compared to the monthly adult dosing regimen currently being studied in clinical trials. A time-to-event model describing the concentration-effect relationship of preventive DHA-PQ administration was used to explore the potential clinical efficacy of once-weekly adult dosing regimens. Loading dose strategies were evaluated and the advantage of weekly dosing regimen was tested against different degrees of adherence. Assuming perfect adherence, three tablets weekly dosing regimen scenarios maintained malaria incidence of 0.2 to 0.3% per year compared to 2.1 to 2.6% for all monthly dosing regimen scenarios and 52% for the placebo. The three tablets weekly dosing regimen was also more forgiving (i.e., less sensitive to poor adherence), resulting in a predicted ∼4% malaria incidence per year compared to ∼8% for dosing regimen of two tablets weekly and ∼10% for monthly regimens (assuming 60% adherence and 35% interindividual variability). These results suggest that weekly dosing of DHA-PQ for malaria chemoprevention would improve treatment outcomes compared to monthly administration by lowering the incidence of malaria infections, reducing safety concerns about high PQ peak plasma concentrations and being more forgiving. In addition, weekly dosing is expected to reduce the selection pressure for PQ resistance.

## INTRODUCTION

Antimalarial drugs are used in various ways to prevent malaria. World Health Organization (WHO)-recommended preventive therapies such as intermittent preventive therapy (IPT) has been used increasingly in recent years especially in young children (IPTc) and pregnant women (IPTp) living in areas where malaria is endemic (1). IPT usually involves giving a curative treatment dose of an effective antimalarial drug at predefined intervals in areas of high malaria transmission to provide both clearance of asymptomatic parasitemia and posttreatment chemoprevention.

Sulfadoxine-pyrimethamine was the first combination for IPT investigated in pregnant women as an alternative to weekly chloroquine chemoprophylaxis (2). The WHO recommends that IPTp-SP is given as part of antenatal care services for pregnant women in moderate to high malaria transmission areas in Africa (3). This is now compromised by worsening drug resistance in many parts of Africa (4, 5), and so dihydroartemisinin-piperaquine (DHA-PQ) has been evaluated as an alternative (6). This artemisinin-based combination therapy (ACT) is widely used in South East Asia and exhibits excellent efficacy and tolerability (2). However, its efficacy has declined recently in parts of the Greater Mekong subregion because of artemisinin and PQ resistance (7, 8). An optimized dosing regimen of combination therapy is an essential part of protecting antimalarials against the development of resistance.

This study aimed to evaluate alternative preventive dosing regimens of DHA-PQ based on extensive simulations with a previously developed population pharmacokinetic-pharmacodynamic (PK-PD) model (9). The simulation framework was used to compare multiple scenarios of adult weekly dosing to the currently practiced adult monthly dosing (i.e., once daily DHA-PQ for three consecutive days, administered once a month). In addition, the benefit of different loading dose regimens and the possibility of reducing the total weekly dose (i.e., two tablets weekly compared to three tablets weekly) were also evaluated. The effects of nonadherence were also investigated since this is a major cause of poor treatment responses (10).

## RESULTS

**FIG 1:**
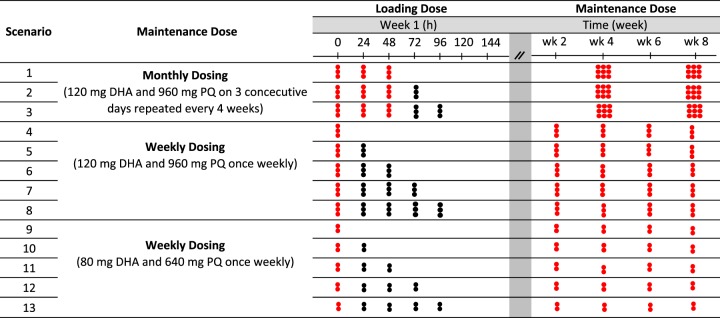
Simulated scenarios for monthly and weekly maintenance dosing regimens (red circles), including different loading dose strategies (black circles).

**FIG 2:**
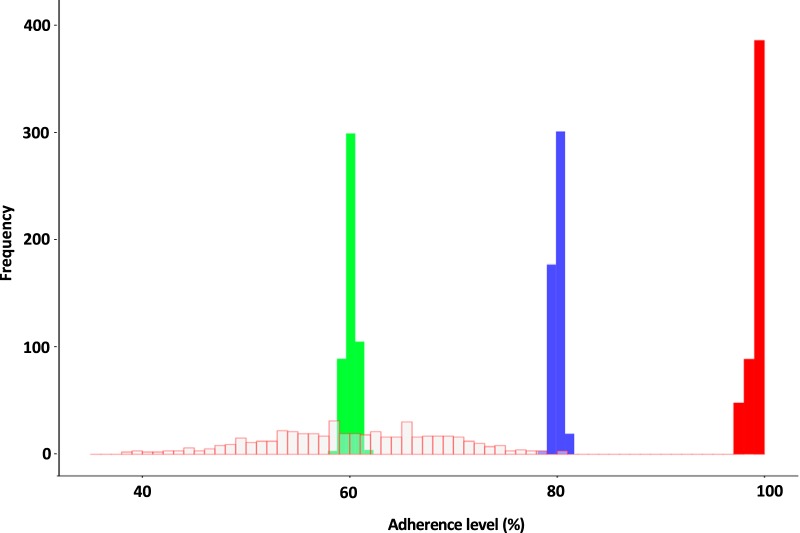
Simulated distribution of individual total patient adherence for 100% adherence (solid red), 80% adherence (blue), 60% adherence (green), and 60% adherence with interindividual variability (open red).

An overview of the dosing in the simulated scenarios is presented in Fig. 1, and the simulated distribution of individual total patient adherence is presented in Fig. 2. Further details about the simulation framework are presented in Materials and Methods.

The predicted malaria mean incidence was 52% per year in patients receiving placebo treatment. The predicted average incidence of malaria was between 0.2 and 0.3% per year for the three tablets weekly adult dosing regimen and between 0.3 and 0.7% for the two tablets weekly dosing regimen compared to 2.1 to 2.6% with the monthly dosing regimens, when complete adherence for all patients was assumed.

Two different assumptions on adherence patterns were evaluated when analyzing standard monthly dosing; the assumption that all three doses were dependent on each other produced an average malaria incidence of 17.2% per year for the three-tablet regimen. This assumption gave higher malaria incidence compared to when the doses were regarded as independent of each other (11.3%). Hence, the more conservative approach of independent dose adherence was investigated further.

**FIG 3:**
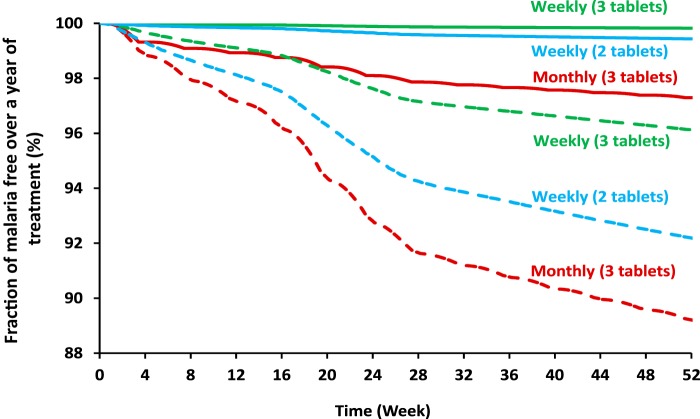
Simulated fraction of malaria free subjects under the assumption of 100% adherence (solid lines) and 60% adherences with 35% interindividual variability (dashed lines), for monthly dosing (red), three tablets weekly dosing (green), and two tablets weekly dosing (blue) over a total treatment period of 1 year.

Nevertheless, three tablets weekly dosing was substantially less affected by poor adherence compared to monthly dosing, but two tablets weekly dosing showed a modest improvement in efficacy compared to a monthly dosing regimen. Reducing the average population adherence by 20%, resulted in yearly malaria incidences of 0.8 to 1.2%, 1.2 to 3.2%, and 4.5 to 5.4% for three tablets weekly dosing, two tablets weekly dosing, and monthly dosing regimens, respectively. A scenario of 60% adherence and between subject variability resulted in yearly malaria incidences of 3.4 to 4.7%, 4.0 to 9.3%, and 9.4 to 10.1% for three tablets weekly dosing, two tablets weekly dosing, and monthly dosing, respectively. Complete incidence results for both monthly and weekly dosing regimens are summarized in Table 1, and a graphical representation of the simulated fraction of malaria-free subjects during 1 year of treatment is presented in Fig. 3.

**TABLE 1:** Predicted mean malaria incidences per year for each dosing scenario at different adherence levels

Dosing schedule^*a*^	Scenario	Yearly malaria incidence (%) at various adherence levels			
		(A: 100%)	(B: 80%)	(C: 60%)	(D: 60% + 30% IIV)
Monthly maintenance	1: No loading dose	2.6	5.4	10.4	10.1
	2: One loading dose	2.4	4.8	9.4	10.2
	3: Two loading doses	2.1	4.5	10.4	9.4
Weekly maintenance (three tablets)	4: No loading dose	0.3	1.2	4.5	4.7
	5: One loading dose	0.2	1.1	3.7	4.3
	6: Two loading doses	0.2	0.9	3.2	3.9
	7: Three loading doses	0.2	0.8	2.8	3.7
	8: Four loading doses	0.2	0.8	3.1	3.4
Weekly maintenance (two tablets)	9: No loading dose	0.7	3.2	8.4	9.3
	10: One loading dose	0.6	2.5	7.7	8.4
	11: Two loading doses	0.6	2.2	7.6	7.8
	12: Three loading doses	0.3	1.3	3.8	4.2
	13: Four loading doses	0.3	1.2	4.5	4.0

a Details for each specific dosing are specified in Fig. 1. Scenario numbers correspond to those listed in Fig. 1.

**FIG 4:**
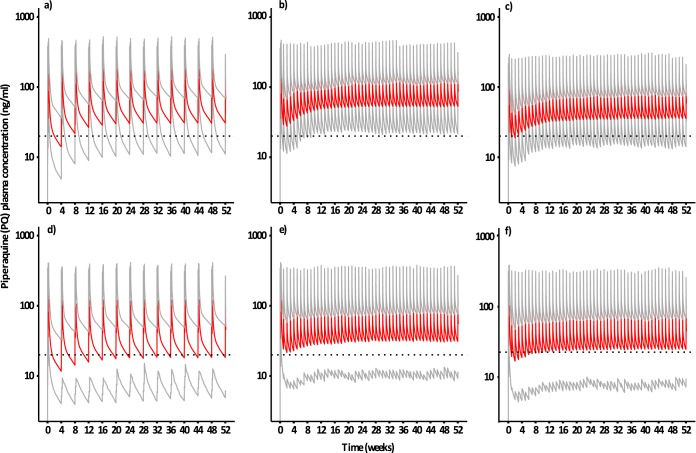
Simulated piperaquine plasma concentration-time profiles when assuming 100% adherence (a to c) and 60% adherences with 35% between subject variability (d to f). Predicted median plasma concentrations (red lines) and and 2.5th and 97.5th percentiles (gray lines) are presented for monthly dosing (a and d), three tablets weekly dosing with two loading doses (b and e), and two tablets weekly dosing with two loading doses (c and f). The dashed horizontal line represents a previously presented IC_95_ value of 20 ng/ml (9).

The predicted steady-state peak concentrations (*C*_max_) were lower for both weekly dosing regimens compared to the monthly dosing, whereas predicted trough concentrations were substantially higher (Table 2 and Fig. 4). However, only three tablets weekly dosing resulted in predicted average steady-state trough concentrations above 20 ng/ml (95% inhibitory concentration [IC_95_] for PQ) at all adherence levels evaluated (9). Three tablets weekly dosing at perfect adherence resulted in predicted steady-state trough concentrations above 20 ng/ml in 95% of simulated patients, whereas all other dosing scenarios showed various degrees of patients with steady-state trough concentrations below the 20-ng/ml cutoff level.

**TABLE 2:** Predicted median piperaquine trough (*C*_min,ss_) and peak (*C*_min,ss_) plasma concentrations at steady state and the first trough concentration (*C*_min,1_) for each dosing scenario at different adherence levels

Dosing schedule^*a*^	Scenario	Piperaquine concn (ng/ml) at various adherence levels											
		(A: 100%)			(B: 80%)			(C: 60%)			(D: 60% + 30% IIV)		
		*C*_max,ss_	*C*_min,ss_	*C*_min,1_	*C*_max,ss_	*C*_min,ss_	*C*_min,1_	*C*_max,ss_	*C*_min,ss_	*C*_min,1_	*C*_max,ss_	*C*_min,ss_	*C*_min,1_
Monthly maintenance	1: No loading dose	236.3	27.5	14.0	214.6	20.8	12.5	186.7	15.4	11.1	192.7	14.8	11.6
	2: One loading dose	231.5	27.5	18.3	215.1	20.8	15.9	188.8	15.6	14.3	190.4	15.0	13.8
	3: Two loading doses	238.1	28.4	22.7	208.2	20.6	19.8	188.4	14.7	17.1	190.2	15.9	16.7
Weekly maintenance (three tablets)	4: No loading dose	218.5	47.9	9.7	208.2	37.3	9.9	185.4	25.8	9.7	189.9	25.1	9.8
	5: One loading dose	219.8	48.4	20.7	203.5	35.9	20.8	190.0	24.3	20.8	188.5	25.0	20.9
	6: Two loading doses	215.1	48.8	33.6	201.6	34.5	31.2	186.1	26.1	28.6	189.4	25.3	28.2
	7: Three loading doses	221.1	47.9	51.5	204.5	36.9	45.9	188.8	26.2	39.3	203.1	27.9	38.4
	8: Four loading doses	218.3	48.8	73.1	202.8	35.9	63.4	190.2	25.4	52.1	188.6	25.3	50.5
Weekly maintenance (two tablets)	9: No loading dose	141.0	31.7	5.9	137.8	24.6	5.6	125.8	16.8	5.7	124.9	16.5	5.7
	10: One loading dose	145.3	32.1	11.8	136.8	24.2	12.3	125.4	16.6	11.9	124.8	16.6	12.0
	11: Two loading doses	146.8	32.9	19.3	136.8	23.9	18.2	125.9	16.2	15.7	124.6	16.9	16.4
	12: Three loading doses	147.7	33.0	28.3	128.3	16.5	20.9	129.7	17.2	20.8	129.7	17.1	20.8
	13: Four loading doses	144.9	32.3	40.1	135.1	24.1	32.5	128.6	17.0	26.9	127.2	16.9	27.4

a Details for each specific dosing are specified in Fig. 1. Scenario numbers correspond to those listed in Fig. 1.

The addition of loading doses to the standard monthly dosing regimen was predicted to increase concentrations prior next dose (i.e., day 28 trough concentration) from 14.0 ng/ml (scenario 1A) to 18.3 and 22.7 ng/ml for scenarios 2 and 3, respectively. This increase translated into a marginal decrease in yearly malaria incidence compared to a standard monthly dosing regimen without loading doses (Table 2). Weekly dosing without any loading dose (scenario 4 and 9) resulted in low initial trough concentrations (i.e., day 7 trough concentrations of 9.7 to 9.9 ng/ml and 5.6 to 5.9 ng/ml for three tablets and two tablets weekly, respectively) for all levels of adherence evaluated. However, trough concentrations accumulated quickly to >20 ng/ml due to the frequent dosing. Thus, additional loading doses to the two tablets weekly dosing regimens had marginal impact on the yearly malaria incidence, i.e., an ∼0.1% decrease compared to when not using a loading dose.

### Power analysis.

**FIG 5:**
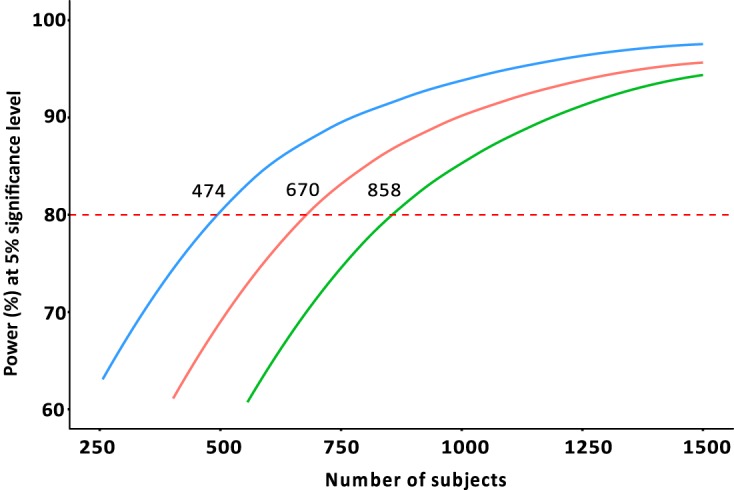
Power curve of the total number of subjects needed for one-sided superiority (red line), two-sided superiority (green line), and noninferiority (blue line) trials. The dashed red line represents the nominal power of 80% needed for a clinical trial.

The power analysis showed that 474 patients (237 in each arm) are required for 80% power with corrected χ^2^ value of 2.7 corresponding to one degree of freedom to conclude that the weekly dosing regimen is not inferior to the monthly dosing regimen (*P* = 0.05). However, for the testing of superiority, almost double the sample size is required for a standard two-sided test (*P* = 0.05). The complete power versus samples size curves are presented in Fig. 5.

## DISCUSSION

Although a monthly DHA-PQ dosing regimen has proved effective and well tolerated in malaria prevention and elimination efforts, we suggest that the regimen can be improved further with more frequent dosing. This would also reduce peak PQ concentrations and hence the risk of associated toxicity. Our results are in line with another recently published study advocating weekly dosing of DHA-PQ based on simulated PQ plasma concentration profiles in a pediatric population (11). This study focused on maintaining PQ plasma concentrations >30 ng/ml under perfect adherence conditions.

This simulation-based study presented here suggests that better treatment outcomes could be achieved with a weekly dosing compared to the current monthly dosing. The three tablets weekly adult dosing was predicted to reduce the malaria incidence 10-fold compared to monthly dosing, assuming perfect adherence and transmission intensity similar to that on the Thai-Myanmar boarder. Two tablets weekly dosing, compared to monthly dosing, resulted in a more modest 3-fold reduction in malaria incidence with assumptions identical to those stated above.

Furthermore, peak PQ concentrations were predicted to be lower at weekly dosing compared to monthly dosing and are hence expected to reduce the concentration-dependent electrocardiographic QT prolongation and any concentration-dependent gastrointestinal adverse effects (12).

Adherence has always been a critical element in ensuring treatment success. In the original trial, where monthly DHA-PQ was compared to bi-monthly DHA-PQ, the dose was given under supervision ensuring high adherence (13). When a treatment is taken outside supervision, it is common that not all patients take their medication as prescribed. This may be more likely in preventive treatment when there are no symptoms to remind the subjects to take their medication. Supervised dosing does ensure good treatment adherence, but it places a logistic and financial burden on health care providers, policy makers, and the patients, especially in rural areas where heath care access is difficult. This limits the implementation of IPT on a wider scale and might prevent an efficacious protection against new malaria infections (14). It is possible that adherence could be increased with simplified dosing regimens such as weekly dosing (15, 16). In addition, the simulations discussed here showed that three tablets weekly dosing is highly forgiving with respect to nonadherence compared to monthly treatment. This could potentially have a large impact on efficacy at a wide scale implementation of IPT.

However, three tablets weekly dosing would result in an increased cost since 33% more tablets are administered compared to monthly treatment. Thus, two tablets weekly dosing (12% lesser tablets compared to standard monthly dosing regimen) was evaluated and showed improved efficacy compared to monthly dosing at all dosing scenarios evaluated, but with a modest improvement at high-level nonadherence. Two tablets weekly dosing resulted in substantially lower peak concentrations compared to all other treatments evaluated and should therefore have an advantageous safety profile compared to other regimens.

Use of loading doses in the first week of treatment expedites reaching steady-state PQ levels. Two additional doses given at 24 and 48 h were needed for weekly dosing regimens in order to quickly reach PQ steady-state concentrations. A loading dose may not be necessary with a weekly dosing regimen. Nevertheless, the increased frequency of DHA's curative effect in weekly dosing might compensate for the delay in attaining PQ steady-state levels, thus a loading dose may not be necessary with a weekly dosing regimen. In addition, a loading dose requirement may complicate the operational implementation of a simple weekly dosing schedule.

The DHA component in this DHA-PQ treatment acts rapidly in reducing the parasite burden, while the PQ component eliminates the residual parasites. Monthly DHA-PQ regimens risk increasing the selection pressure on PQ resistance, especially if adherence is poor, by generating subtherapeutic PQ concentrations before the next dose (17). Weekly dosing reduces the selection pressure by increasing trough PQ concentrations, but it is also expected to reduce the risk of *de novo* resistance because of shorter intervals between curative DHA exposures (17).

The simulations described here are not intended to mimic perfectly the biological variability for a specific population but are rather focused on the relative comparison between different dosing regimens with regard to malaria prevention. Different alternative assumptions regarding the malaria transmission intensity and/or pharmacokinetic characteristics are expected to alter the absolute outcome of the simulations but not the relative comparison of the investigated dosing regimens. However, the power/sample size calculations are sensitive to the underlying malaria transmission and in preparation of an actual study the power calculations should be updated based on the best estimates of the malaria transmission intensity in the region where the study is to be conducted.

In conclusion, the presented simulations suggests that weekly dosing for DHA-PQ malaria chemoprevention would be preferable to monthly dosing to improve malaria chemoprevention by lowering the incidence of malaria infections, reducing safety concerns driven by high PQ peak plasma concentrations, and reduce the sensitivity to poor adherence. In addition, weekly dosing is expected to reduce selection pressure for drug resistance.

## MATERIALS AND METHODS

### Population pharmacokinetic-pharmacodynamic model.

This simulation study was based on data obtained from a clinical trial (ISRCTN65524939) conducted in northwestern Thailand, which included 1,000 healthy adult males whose occupation put them at high risk of malaria (13). The study compared prophylactic efficacy of monthly versus bimonthly DHA-PQ treatment given over a 9-month period. The volunteers were randomized to receive a standard 3-day treatment each month, every other month or an identical placebo treatment, with or without fat.

A PK-PD model was developed to describe the occurrence of malaria infections in the different treatment arms in this study (9). This final concentration-effect relationship model consisted of three disposition compartments with five transit absorption compartments and a time-to-event (TTE) model. The TTE model described the hazard of having a malaria infection, including seasonal variations in the hazard (described by surge functions in the model). This seasonal variation reproduces the monsoon season in this region, in which transmission increases.

The mixture probability implemented on the baseline hazard described individual hazards in relation to age and immunity. The predicted plasma PQ concentrations modulated the hazard of having malaria infection via an inhibitory maximum-effect (*E*_max_) model. PQ plasma concentrations of 6.7 and 20 ng/ml were found to reduce the hazard of having malaria infection by 50% (IC_50_) and 95% (IC_95_), respectively (9). During the brief DHA exposure, cumulative hazard was modulated to represent the lowered risk of developing malaria.

This model was used for translational dose-optimization simulations in children, pregnant women and areas where PQ resistance might arise. The model development has been presented in detail in a previous publication (9). The same model was used in this simulation study to explore the predicted efficacy of a novel weekly dosing.

### Simulation design.

In this simulation study, weekly DHA-PQ dosing regimens consisting of three tablets of DHA-PQ once a week or two tablets of DHA-PQ once a week were compared to the standard monthly adult dosing regimen (i.e., three tablets of DHA-PQ daily for 3 days, once every 4 weeks) (18). A total of 1,000 hypothetical adult subjects who were 31 years of age and 50 kg in body weight were simulated for a period of 1 year for each dosing scenario (Fig. 1) and respective adherence level. The sensitivity to adherence was explored systematically using various levels of adherence (Fig. 2), i.e., perfect adherence (scenario A), 80% adherence (scenario B), and 60% adherence (scenario C) for all dosing regimens. Adherence was quantified here as the proportion of full doses taken correctly throughout the study period, i.e., 80% adherence indicates that 80% of the total doses were taken throughout the study period. DHA-PQ tablets (i.e., either two or three tables) taken daily are assumed one dose and was considered the “unit” for adherence. The cumulative or average adherence probability (*P_j_*) was fixed for each patient throughout the study, according to the different adherence levels.

The dichotomous outcome of the adherence (*X_i_*), generated for each dose, was determined according to a binomial distribution using the inverse transformed technique (19). If *U_i_* … *U_n_*, where *U* is an independent randomly generated number following a uniform distribution (i.e., “0, 1”), Equation 1 then:

(1) $$X_{i}=\left\{ \begin{array}{c} 1,\text{ adherent }\left(\text{if }U_{i}\leq P_{j}\right)\\ 0,\text{ nonadherent }(\text{if }U_{i}>P_{j}) \end{array}\right.$$

Each patient's cumulative adherence is determined as an average of *X_i_*.

The various degrees of adherence explored above assumed every patient to act similarly, i.e., similar average adherence level or no between subject variability, which is not true in reality. To mimic a more realistic clinical adherence setting, wider variability between adherent/nonadherent subjects was included. Between subject variability (η) of 35% was added to the lowest adherence level, i.e., 60% adherence (scenario D) as defined below:

(2) $$\pi \;=\;\exp\;\frac{\left(\log\text{it }P_{j}\;+\;\eta \right)}{\left(1\;+\;\exp\left(\log\text{it }P_{j}\;+\;\eta \right)\right)},\;\eta ∼N\left(0,\omega^{2}\right)$$

where π is the adherence probability and η was assumed to be independent and symmetrically distributed with zero mean and variance ω^2^.

This allowed patients to have their own unique degree of adherence. A more detailed visualization of these scenarios is shown in Fig. 2. In addition, the first dose is usually observed and therefore set to perfect adherence (i.e., 100% adherence) in all dosing scenarios. For monthly dosing regimens, two possible assumptions on adherence were tested: (i) all three daily doses during a month were regarded as dependent on each other, and the adherence was evaluated collectively, i.e., either all three doses were missed or the patient was fully adherent on that particular month, and (ii) the three daily doses during a month were regarded as independent of each other, and the adherence was evaluated individually for each dose.

The pharmacodynamic effects from the simulated scenarios were evaluated through mean disease free survival over 1 year (SUR). The survival, *S*(*t*) (equation 4), an inverse function of the cumulative hazard, *h*(*t*), describes the probability of not having at least one event (i.e., malaria infection) until the end of study time, *t*. Thus, a predicted decrease in the PQ plasma concentration (e.g., nonadherence) resulted in an increased cumulative hazard of presenting a malaria infection.

(3) $$\text{SUR}=\;\frac{1}{n}\sum_{i=1}^{n}S_{i}\left(t\right)$$

(4) $$S\left(t\right)=\;{\text{e}}^{-\int_{0}^{t}h\left(t\right)dt}$$

The predicted PQ concentrations for all simulated scenarios were evaluated by visualization of peak concentrations and trough concentrations during the dosing interval. The second layer of complexity studied was the simulated effects of different loading doses. PQ has a very long terminal elimination half-life (*t*_1/2_ ∼ 20 to 30 days) and *in vivo* drug concentrations will therefore accumulate with repeated dosing in IPT (13). PQ plasma concentration is expected to reach a steady state in approximately 12 weeks with regular dosing. Thus, additional daily doses during the first days of dosing were evaluated to mitigate lower exposure at the beginning of treatment. The exact timing of these additional doses is summarized in Fig. 1.

### Power analysis.

The final pharmacokinetic model was simplified in the power analysis by replacing the transit absorption model with a first-order absorption model (due to computationally long run times with the original model). After verifying that this did not change the PK-PD parameter estimates substantially, this model was used to simulate a randomized clinical trial of 1,000 patients based on the average population of the original study on the Northwest border of Thailand (13). The simulated study had two parallel study arms comprising (i) standard monthly dosing (Fig. 1, scenario 1) and (ii) weekly dosing with the addition of two loading doses at 24 and 48 h (Fig. 1, scenario 6) for a total study duration of 12 months (18). All patients were assumed to have perfect adherence.

A simulation-based power analysis was conducted to investigate the necessary sample size for a study with the aim of concluding that a weekly dosing regimen is noninferior to a monthly dosing regimen or concluding superiority of a weekly dosing regimen over a monthly dosing regimen (20).

To achieve these two aims, hypothesis testing was conducted using a constant hazard TTE model with groupwise comparison between the two treatment arms using a likelihood ratio test (i.e., based on the difference in objective function value [ΔOFV] computed by NONMEM) between the two nested models. The yearly incidence of malaria was the clinical endpoint of the drug effect from these drug regimens.

The sample size needed to achieve the desired 80% power, at a significance level of 0.05, was calculated using a Monte Carlo mapped power (MCMP) approach (20). The MCMP method fits two competing pharmacometric models (i.e., reduced and full model) to simulated data and calculates the difference in individual OFVs (ΔiOFV) between the two models (equation 5). The power to detect a significant difference between groups is then calculated by evaluating the total sum of ΔiOFV at different study sizes (equation 6).

(5) $$\Delta \text{iOFV}=\;{\text{iOFV}}_{\text{REDUCED}}\;-\;{\text{iOFV}}_{\text{FULL}}$$

(6) $$\text{POWER}=\;\frac{\sum_{n=1}^{N}\left(\Delta \text{OFV }\geq \;X_{0.05}^{2}\left(\text{df}\right)\right)}{N}$$

For noninferiority testing, the drug effect in the reduced model was fixed to the chosen noninferior margin. This corresponds to the null hypothesis of monthly dosing (control regimen, C) producing a higher efficacy compared to weekly dosing (test regimen, T). For the full model, the drug effect was estimated to favor the alternative hypothesis. For superiority testing, the drug effect was fixed to zero in the reduced model to indicate no difference between the two treatments, and the drug effect in the full model was estimated to favor the alternative hypothesis.

### Type I error analysis.

A stochastic simulation and estimation (SSE) approach with 10,000 replicates was used to deflate the type I error rate. A reduced model was used to simulate 1,000 patients with two arms, i.e., monthly dosing (*n* = 500) and weekly dosing (*n* = 500) to favor the null hypothesis. The data were fitted to both the full and reduced model. The ΔOFV output of each individual replicate was calculated and ranked in descending order and the fraction which provided a significant difference that corresponds to 0.05 (the 95th percentile) was obtained as the nominal OFV cutoff. The new χ^2^ value was later used to recalculate the power and sample size from the MCMP method for the noninferiority trial.

### Software.

Drug concentration and efficacy data simulations were performed with Berkeley Madonna (21), and postprocessing and plots were prepared using R studio version 2.15.3 (22, 23). Stochastic simulation and estimation was performed using nonlinear mixed-effects modeling in the software NONMEM, v.7.3 (24) (ICON Development Solutions, Ellicott City, MD), facilitated by a routine developed in PsN (v.4.5.2) (25).
